# Hypertonic saline enhances the efficacy of aerosolized gentamicin against *Pseudomonas aeruginosa*

**DOI:** 10.1038/s41598-020-61413-4

**Published:** 2020-03-09

**Authors:** Hui-Ling Lin, Li-Chung Chiu, Gwo-Hwa Wan, Chung-Chi Huang, Zong-Tian Lee, Yun-Tzu Lin, Shan-Rong Wu, Chi-Shuo Chen

**Affiliations:** 1 Department of Biomedical Engineering and Environmental Sciences, National Tsing Hua University, Hsinchu, 30013 Taiwan; 2 Department of Respiratory Therapy, Chang Gung University, Taoyuan, 33302 Taiwan; 3 Department of Respiratory Therapy, Chang Gung Technology University, Chiayi, Taiwan; 4 Department of Respiratory Therapy, Chiayi Chang Gung Memorial Hospital, Chiayi, 61363 Taiwan; 5 Department of Thoracic Medicine, Chang Gung Memorial Hospital, Chang Gung University College of Medicine, Taoyuan, 33301, 61363 Taiwan; 6 Department of Obstetrics and Gynecology, Taipei Chang Gung Memorial Hospital, 10501 Taipei, Taiwan; 7 Department of Respiratory Therapy, Cheng Hsin Hospital, Taipei, 11201 Taiwan

**Keywords:** Antimicrobials, Medical research

## Abstract

Aerosol inhalation is a promising strategy for the delivery of antibiotic agents. The efficacy of antibiotic treatment by aerosol inhalation is reduced by the formation of microbial biofilms in the respiratory system and excessive airway mucus build-up. Various approaches have been taken in order to overcome this barrier. In this *in vitro* study, we used hypertonic saline (7%, by weight), a low cost Food and Drug Administration-approved reagent, as an aerosol carrier to study its effects with the antibiotic, gentamicin, on the most common respiratory opportunistic pathogen, *Pseudomonas aeruginosa*, present in the mucus. The results indicated that the hypertonic saline aerosol containing gentamicin, a low cost antibiotic, significantly eliminated biofilm growth by ~3-fold, compared to the regular saline aerosol containing gentamicin. In addition to enhancing the penetration efficiency of drug molecules by 70%, bacterial motility also decreased (~50%) after treatment with aerosolised hypertonic saline. In conclusion, our results demonstrate that hypertonic saline can significantly enhance the efficacy of antibiotic aerosols, which may contribute to the current use of inhaled therapeutic compounds.

## Introduction

Antibiotic agents are broadly applied in the treatment of various respiratory diseases, such as acute respiratory infections, pneumonia, acute exacerbation of chronic obstructive pulmonary disease, secondary infections, and cystic fibrosis^1^. The annual consumption of antibiotics is estimated to have substantially increased from a daily defined dose of 21.1 billion in 2000 to 34.8 billion in 2015^2^. To effectively treat bacterial pneumonia, the antibiotics must achieve the minimum inhibitory concentration (MIC) in the lungs. When antibiotics are administered by intravenous injection, the concentration of antibiotics in the lungs depends on the drug formulation and delivery efficiency. Poor drug penetration into the infected site in the lungs may result in the need for increased therapeutic dosage with higher incidence of adverse effects^3,4^. Recently, the administration of medication to treat pulmonary diseases via inhalation therapy has gained interest; the foremost advantage of inhaled antibiotics is that they can directly increase the concentration of the therapeutic compound at the target site. In addition, inhaling aerosolized drugs can minimize systemic adsorption and toxicity^5^.

Although the concept of directly delivering antibiotic agents to the infected sites is highly promising and desirable, certain challenges, such as determining the inhalation formulations and designing the delivery devices, need to be overcome to optimize aerosolized antimicrobial treatments. One of the main challenges in delivering inhaled antimicrobial peptides is the inhibitory effect of sputum. Sputum is composed of mucin proteins, derived DNA, inflammation mediators, bacteria, and filamentous actins; it serves as the first physical protective barrier against exogenous agents, such as bacteria and pollutants, in the respiratory system^6^. Mucin proteins bind to antibiotics, thereby reducing the concentration of freely diffused active antibiotics^7^. Thus, physiological efficacy in the respiratory system can be highly restricted by the penetration of antibiotics within the mucus layer^8,9^. Moreover, active muco-ciliary clearance, an innate defense system that cleans exogenous debris, can sufficiently remove the antibiotic aerosols deposited in the mucus leading to a reduction in the concentration of the deposited antibiotic^5^.

In addition, with regard to hindered delivery of antibiotics to the infection sites, mucus is closely related to another challenge in aerosolized antibiotic treatment, i.e., the formation of microbial biofilms. For instance, the antibiotic-resistant biofilm that forms in the airways in cystic fibrosis, is facilitated by thick and dehydrated mucus^10^. *In vitro* experiments have also demonstrated that mucin–microbial interactions facilitate biofilm formation and antibiotic resistance^11^. Numerous studies have demonstrated that the bacteria within a biofilm are highly resistant to antibacterial agents and are 10–1,000 fold less susceptible to antimicrobial agents than those in a free-floating culture; thus, the bacteria residing deep within a biofilm are not always eliminated^12^.

To enhance the efficacy of aerosolized drugs, various approaches are being developed to increase drug penetration^5^. Considering the cost and potential side effects, we aimed to investigate the effect of a hypertonic saline aerosol used as an antibiotic carrier. Hypertonic saline aerosols have been used clinically to treat patients with cystic fibrosis and children with acute bronchiolitis^13,14^. Kellett *et al*. investigated the long-term effects of hypertonic saline on patients with bronchiectasis and found that the patients treated with inhaled hypertonic saline (7%, by weight) had ameliorated pulmonary function, health-related quality of life, mucus viscosity, and airway clearance, compared to inhaled normal saline^15,16^. In addition to thinning the viscosity of mucus, hypertonic saline can be used as a non-antibiotic treatment to inhibit microbial growth because of its high osmotic pressure^17^. Havasi *et al*. reported that high concentrations of saline mixed in an agar culture can block *Pseudomonas aeruginosa* from spreading and reduce bacterial density^18^. However, because aerosol deposition varies depending on the devices and generators used to emit the dose, the influence of aerosolized hypertonic saline remains to be evaluated. Moreover, the compounding effects of hypertonic saline and antibiotic have not yet been fully explored. In this study, we used *P. aeruginosa*, one of the primary pathogens in acute and chronic airway infection in cystic fibrosis and bronchiectasis^19^, cultured on a mucin matrix to evaluate the inhibitory effects of a hypertonic saline/antibiotic aerosol and its underlying mechanism.

## Results

### Suppression of bacterial growth by hypertonic-gentamicin aerosols

**Figure 1:**
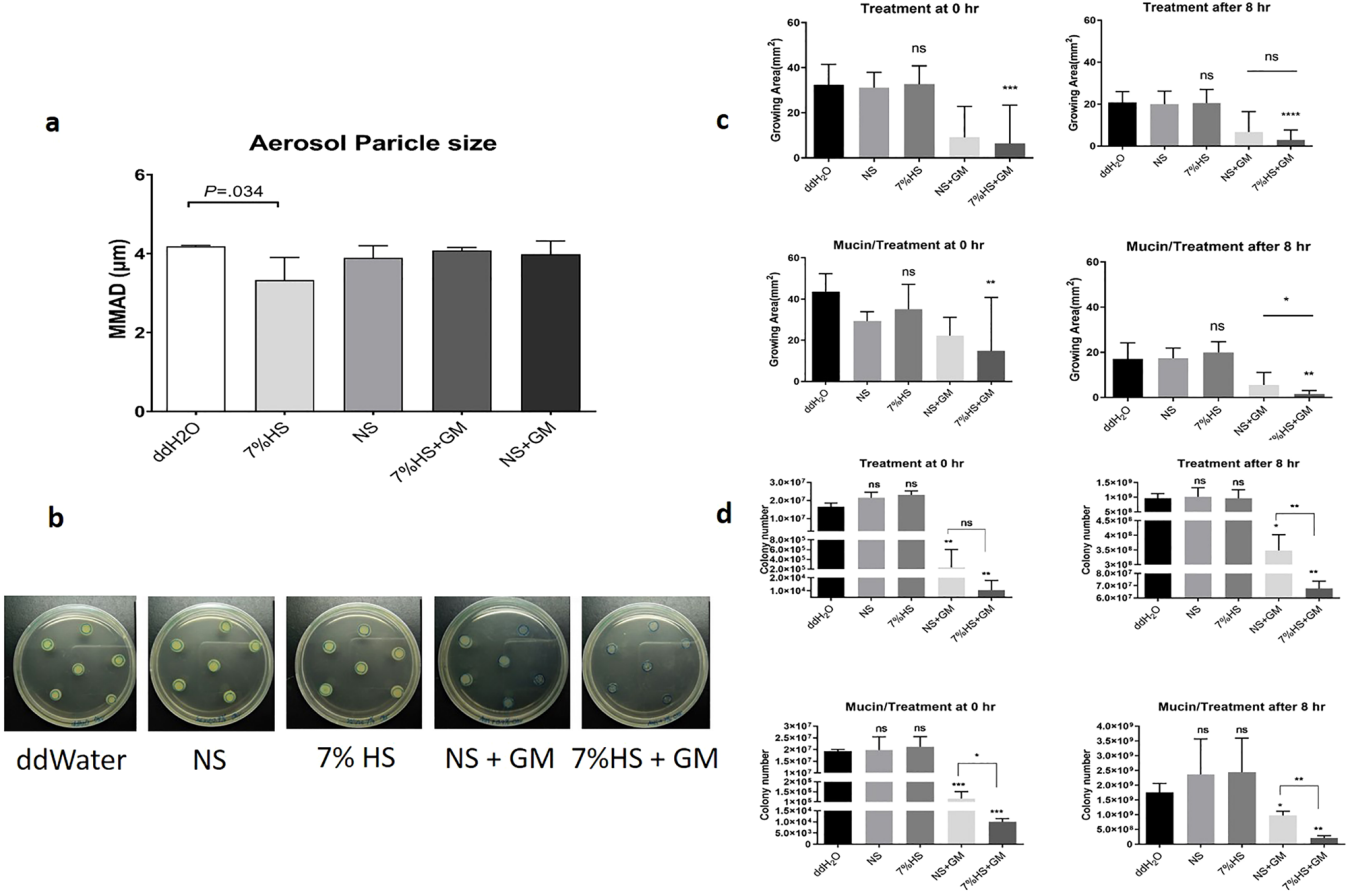
*Pseudomonas aeruginosa* (PAO1 strain) with aerosol treatment. (**a**) The diameter distribution of aerosols in different reagents. (HS: hypertonic saline, NS: normal saline, GM: gentamicin, VMD: volume median diameter.) (**b**) Images of *P. aeruginosa* colonies treated with aerosols after 24 h incubation. (**c**) Quantitative analysis of colony diameter after treatment with aerosols. The growth area was used to evaluate the bacterial susceptibility to the aerosol treatments; the data shows that hypertonic saline can facilitate the inhibitory efficacy of gentamicin. N > 20, data collected from three independent experiments, and all data were presented as mean ± SD. (**d**) Quantitative analysis of survival bacteria after treatment. *p  <  0.05, **p  <  0.01, ***p  <  0.001.

Effective deposition at the infection site is essential for aerosol treatment. With the recognition of the potential of hypertonic saline, we evaluated the effects of aerosolized hypertonic saline with gentamicin. In this study, we first evaluated the diameter of a nebulizer-generated aerosol under different experimental conditions. The measurement of hypertonic saline was 3.5 ± 0.3 µm, significantly different from ddWater, yet without clinical significance; moreover, the obtained diameter (4 ± 0.3 µm) in Fig. 1a was expected to be suitable for aerosol treatment.

After confirming consistent aerosol size distribution in the experiments, *P. aeruginosa* colonies were prepared on agar plates with or without a mucin matrix, followed by treatment with different aerosols in Fig. 1b. To evaluate the influence of microbial colonization on drug tolerance, we treated *P. aeruginosa* at 0 h and 8 h after plating and monitored the bacterial growth at 24 h after treatment. In the treatment group at 0 h and 8 h, the substrate without mucin revealed no obvious alterations in the colony formation (*p* = 0.872 and *p* = 0.997, respectively), in both isotonic (normal) saline (31.1 ± 6.8 mm^2^) and hypertonic saline (32.7 ± 8.1 mm^2^) aerosol treatment groups, compared to the control group (ddWater, 32.4 ± 9.0 mm^2^) (Fig. 1c). We also evaluated the compounding effects of hypertonic saline and an antimicrobial agent, gentamicin. We found that, on the substrate without a mucin matrix, both saline-gentamicin and hypertonic-gentamicin aerosols effectively limited colony growth (*p* < 0.001). In addition to measuring colony size, we performed the colony formation assay to evaluate whether aerosolized hypertonic saline can enhance the gentamicin efficacy (Fig. 1d). Under our experimental setting, there were no significant differences between normal saline aerosol, hypertonic saline aerosol, and control group (*p* = 0.969). Gentamicin showed better efficacy with hypertonic saline aerosol than with the saline aerosol (*p* < 0.001); though the difference was not obvious while treating bacteria at 0 hr. Significantly more bacteria survived with saline-gentamicin treatment than hypertonic-gentamicin aerosol at 8 hr (*p* < 0.01). We evaluated the efficacy of the hypertonic-gentamicin aerosol on *P. aeruginosa* grown on a mucin matrix. When compared to the bacterial growth on substrate without mucin, the diameter of *P. aeruginosa* colonies showed approximately 1.3-fold increase in control groups in first 8 hr culture (treatment at 0 hr and incubated for 8 hr before measurement), 43.6 ± 2.5 mm^2^ with mucin, and 32.4 ± 2.6 mm^2^ without mucin, p = 0.0054. No significant difference was found between the control and hypertonic saline after treatment at 0 h (*p* = 0.179); furthermore, there was no significant difference with the antibiotic treatment after treatment at 8 h (*p* = 0.264; Fig. 1c). In addition, data showed that the colony size decreased to approximately 50% after treatment in the presence of mucin, compared to 20% without mucin (treatment at 0 h); the data from the colony formation assay showed that more bacteria survived on a mucin matrix than without mucin matrix after saline-gentamicin aerosol treatment (mucin: no mucin = 0.6%: 0.2%). Notably, in the presence of the mucin matrix, the hypertonic-gentamicin aerosol suppressed colony growth more effectively than the saline-gentamicin aerosol (*p* = 0.024). We observed only 0.05% survival with hypertonic-gentamicin aerosol and 0.6% with saline-gentamicin aerosol (*p* < 0.01; Fig. 1d, mucin matrix with treatment at 8 h) where treatment was given after 8 h of bacterial growth. The saline-gentamicin treatment achieved a 4-fold reduction in colony size (17.1 ± 4.5 mm^2^ to 5.5 ± 5.5 mm^2^, *p* < 0.001; treatment after 8 h) and the hypertonic-gentamicin aerosol achieved a 10-fold reduction (17.1 mm^2^ to 1.5 ± 1.5 mm^2^, *p* < 0.001; treatment after 8 h). Our data indicated that mucin matrix increased the growth and gentamicin tolerance of *P. aeruginosa*. Hypertonic saline aerosols promoted the microbial inhibitory efficacy of gentamicin, particularly in the presence of a mucus matrix.

### Hypertonic saline enhances drug diffusivity within mucin matrix

**Figure 2:**
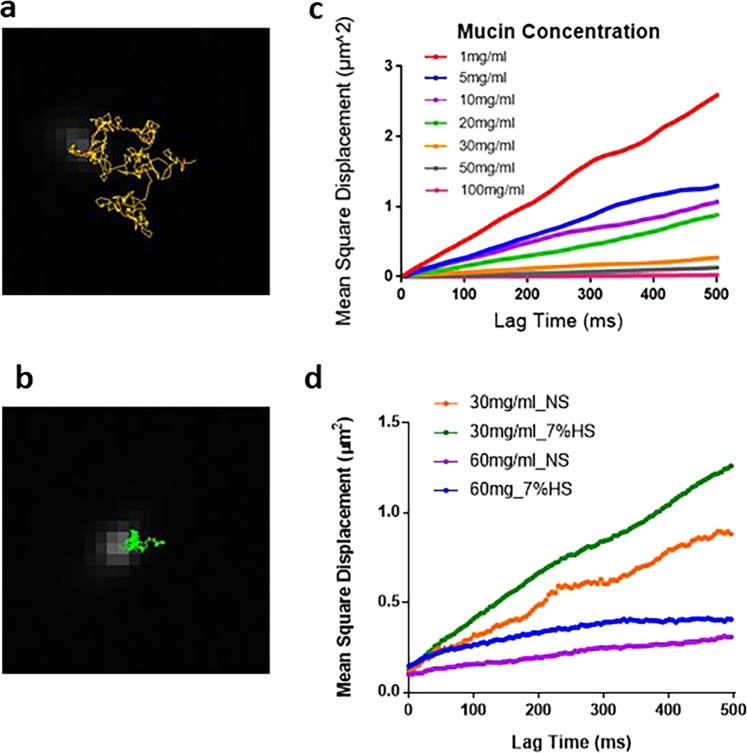
Diffusivity evaluation with particle tracking analysis. The representative trajectory of nanospheres in the mucin matrix (**a**: low-concentration mucin; **b**: high-concentration mucin). The Brownian diffusion of nanospheres was hindered by the mesh of the mucin matrix when the concentration was higher. (**c**) The average mean square displacement (MSD) of nanospheres in saline (0.9 wt%) with different concentrations of mucin. Results show that MSD decreased with increasing mucin concentration. (**d**) The averaged MSD of nanospheres within the mucin matrix in saline and hypertonic saline (7% wt%) with physiologically relevant mucin concentrations of 30 mg/mL and 60 mg/mL. Data was collected form three independent experiments, N > 5 for each experimental condition.

The dry weight of mucus^20^, to represent the polymeric mucus matrix and studied the alteration of drug diffusivity after hypertonic saline treatment. To avoid variations resulting from heterogeneous aerosol deposition, we evaluated the mucus matrix directly in hypertonic saline. First, we compared the Brownian motion of nanospheres within the entangled mucin matrix at different concentrations; a lower mean-square displacements (MSDs) indicated lower diffusivity. A lower MSD of microspheres was observed with increasing mucin concentrations from 1 mg/mL to 100 mg/mL (*p* < 0.001), which indicated the range of physiological conditions (Fig. 2a–c). We then compared the MSD of nanospheres within the mucin matrix with hypertonic saline. Data indicated a higher MSD of nanoparticles when mucin was dissolved in the hypertonic saline. The measurements indicated that hypertonic saline increased the nanosphere diffusivity in the mucin matrix (*p* < 0.001), as shown in Fig. 2d, which implies that hypertonic saline can be applied to disperse entangled mucin polymeric chains.

**Figure 3:**
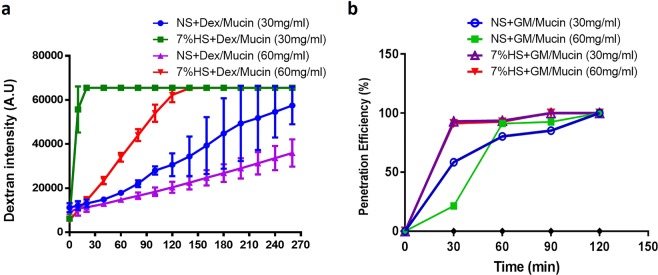
Dextran and Gentamicin diffusion through the mucin matrix. (**a**) Mucus matrices of different concentrations laid in the apical compartment of a transwell support were treated with fluorescence labelled-dextran in saline or hypertonic saline. Data shows that hypertonic saline can facilitate the dextran penetration through mucin matrix. Data was collected from three independent experiments. (**b**) We observed hypertonic saline-gentamicin in both concentrations of mucin reached 100% penetration efficiency at 30 min, compared to gentamicin with saline, which took 60 min in 60 mg mucin and 120 min in 30 mg mucin.

To confirm that hypertonic saline can facilitate antibiotic penetration through mucus, we also quantified the molecule penetration using transwell membranes and fluorescence-labelled dextran. Fluorescent dextran dissolved in either saline or hypertonic saline was gently added on to the top of the mucin matrix, and the dextran that penetrated through the mucin matrix was collected from the bottom of the well. The data showed that the polymeric layer with a higher concentration of mucin hindered the dextran penetration (Fig. 3a). Notably, dextran that was dissolved in hypertonic saline showed better penetration efficiency compared to normal saline. We carried out the penetration assay with gentamicin and quantified the penetration of gentamicin with liquid chromatography–mass spectrometry analysis. Similar to the results of dextran, a lower penetration of gentamicin was observed when there was a high concentration of mucin (Fig. 3b). Under these experimental conditions, due to the molecular weight of gentamicin being less than dextran, we found gentamicin penetrated more efficiently and reached saturation after 2 h in all the groups. The experimental data shows that a high-concentration mucin matrix (60 mg/mL) is sufficient to hinder the molecular penetration. Dextran and gentamicin in hypertonic saline can efficiently penetrate the mucin matrices at both 60 mg/mL and 30 mg/mL.

### Alteration of colony microstructures by the hypertonic antibiotic aerosol

**Figure 4:**
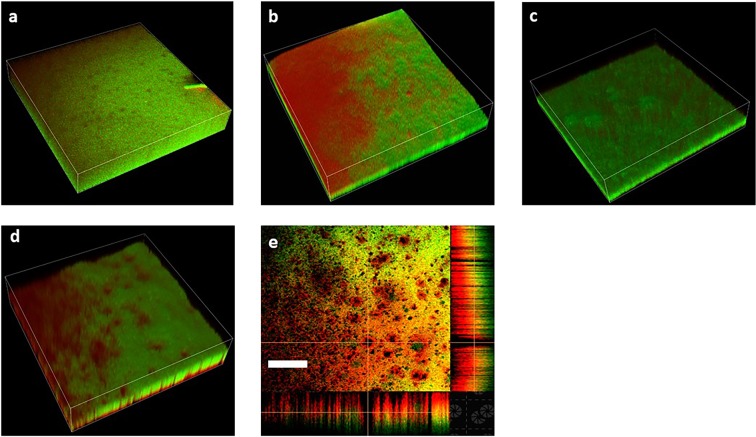
3-D images of colony microstructure and distribution of live and dead bacteria on the biofilm structure after aerosol treatments. Live cells are pseudo-colored green, and dead cells are pseudo-colored red. (**a**) Overnight growth of the *Pseudomonas aeruginosa* (PAO1 strain) colony. (**b**) The colony treated with the saline-gentamicin aerosol. (**c**) Cells showed patch-like structures after hypertonic saline aerosol treatment. (**d**) Distinct holes in the colony after hypertonic saline-gentamicin aerosol treatment. (**e**) Optical section of the colony treated with the hypertonic saline-gentamicin aerosol. (Scale bar: 50 µm).

To understand the influence of an aerosol deposited on a microbial colony, we studied the microstructure of a *P. aeruginosa* colony using a laser scanning confocal microscope. The control group (ddWater) had no distinguishable features and the live and dead bacteria were distributed homogeneously within the colony as shown in Fig. 4a. The saline-gentamicin aerosol group, we observed dead bacteria accumulated in certain regions; most live bacteria were located in the other regions (Fig. 4b). We speculated that the segregated microbial distribution was due to the heterogeneous aerosol deposition. The deposited antibiotic reached the minimal inhibitory concentration, which could abolish the bacterial growth only in certain regions. Similar bacterial segregation was found with hypertonic saline aerosol treatment (Fig. 4c). Hypertonic saline-aerosol showed antimicrobial activity as well. Distinguished microbial clusters were observed after hypertonic saline aerosol treatment. Bacterial growth was limited in specific areas, compared to a homogenous biofilm in the control group. Notably, micron-size holes were observed in the microbial colony after hypertonic-gentamicin aerosol treatment; no bacterial growth occurred within them even after 8 h of incubation following treatment (Fig. 4d). We also observed a unique rim structure surrounding the hole, in which dead bacteria had accumulated (Fig. 4e). Analogous to the diffusion disk test, the accumulation of dead bacteria indicated the efficacy of the hypertonic-gentamicin aerosol.

### Hypertonic-saline aerosol eliminates microbial motility

**Figure 5:**
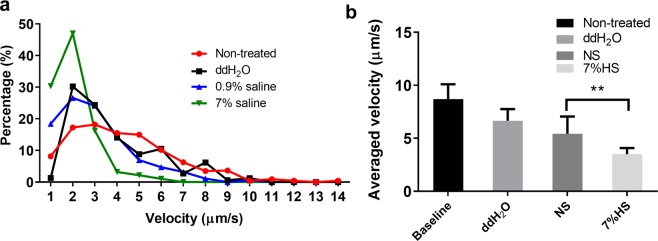
Influence of deposited saline aerosols on bacterial swimming velocity. (**a**) Data represents the population ratio of swimming velocity with different aerosol treatments. (**b**) The average velocity of bacteria. Data is presented as mean ± SD from 3 independent experiments. ANOVA analysis with Bonferroni post hoc test was applied and indicated as **p  <  0.001.

*P. aeruginosa* was mixed in the mucin (30 mg/mL) to mimic bacterial swimming within the mucus layer of the airway; the swimming track was monitored using a time-lapse microscope immediately after treatment with hypertonic-saline aerosols at different concentrations (Fig. 5a). Within the mucin matrix, *P. aeruginosa* showed a broad spectrum of swimming velocities, ranging from 2–20 µm/s. No obvious difference was found between aerosolized ddWater (8.7 ± 1.4 μm/s) and normal saline treatments (5.4 ± 0.6 μm/s), both of which slightly reduced the bacterial swimming velocity. Compared to aerosolized saline, aerosolized hypertonic saline further reduced the bacterial swimming velocity to 3.51 ± 0.58 μm/s (Fig. 5b; *p* < 0.001).

## Discussion

Clinical reports have indicated that excessive mucus accumulation in the airway is associated with chronic inflammation; moreover, hypertonic saline aerosols have been used to treat patients with cystic fibrosis or children with acute bronchiolitis^13,14^. Previous studies have shown that a hypertonic saline aerosol can reduce mucus viscosity and facilitate the removal of mucus from a patient’s airways, resulting in reduced frequency of pulmonary exacerbation^20^. The long-term effects of hypertonic saline on patients with bronchiectasis were investigated in patients treated with inhaled hypertonic saline; these patients showed a better health-related quality of life than patients treated with inhaled normal saline^15,16^.

Herein, to further expand the applications of hypertonic saline in respiratory therapies, we have demonstrated the compounding effects of hypertonic saline aerosols and an antibiotic agent in our *in vitro* experiments and have explored the mechanisms contributing to the therapeutic efficacy. Although various supplementary agents have been developed to promote drug penetration or reduction in airway mucus viscosity, concerns regarding alterations in aerosol size hinder their application^21^. Appropriate aerosol size is critical for efficient deposition in the respiratory system. Our data showed that hypertonic saline does significantly change the aerosol size, only 0.5 µm different comparing to other conditions, thus indicating that it can serve as an aerosol additive without reducing the aerosol deposition efficiency.

Michon *et al*. reported the antimicrobial activity of hypertonic saline as an MIC_50_ of 4%–6% in saline-agar or saline-broth assay^17^. In comparison to the continuous osmotic stress provided in saline-agar or saline-broth assays used in previous studies, bacterial colonies were exposed to limited osmotic pressure changes with single aerosol treatment in our experiment. Based on these results, we noticed 7% hypertonic aerosols inhibited the bacteria growth in microscale alone but would not eliminate colony growth in the colony assays. The outcome indicated that deposited hypertonic saline aerosols may generate osmotic stress locally, which can eliminate the bacterial growth^22^. However, the total amount of deposited aerosols was insufficient to change the salinity throughout the environment to reach the minimum inhibitory concentration. This result not only highlighted the importance of aerosol deposition but also demonstrated the complexity of aerosol treatments.

In addition to the antimicrobial activity caused by osmotic stress, two potential mechanisms were speculated to contribute to the enhancement of gentamicin efficacy: (1) hypertonic saline can decrease the hindrance of mucins to antimicrobial agents and (2) hypertonic saline can limit microbial migration, which restricts the bacteria within the high antibiotic concentration region near the deposited aerosol.

We first tested the influence of hypertonic saline on gentamicin diffusion within the mucin matrix. Measuring the trajectories of nanospheres, we found longer MSDs of nanospheres in the hypertonic saline-treated mucins. The longer MSD implied that the hypertonic saline may lose the entangled mucin matrix and increase the potency of drug penetration in the mucus. The results conformed to the proposed polymeric model, which indicated that the negatively charged mucin polymeric chains (10–40 MD) entangle with each other through electrostatic interactions with positive divalent ions, such as Ca^++^ and Mg^++^ ^9^; the hypertonic saline can disrupt ionic interactions within the mucus gel and reduce the cross-linking of the entangled mucin polymers, which can reduce the viscosity of the mucus^13^. Excessive potassium ions in hypertonic saline may compete with the divalent ions and lead to a decrease in the entanglement between mucin polymeric chains. Moreover, the increased diffusion of drug molecule in loosening mucin matrix was confirmed using fluorescent dextran and the LC-MS. Data supported the MSD findings and further showed that hypertonic saline can promote antibiotic penetration by loosening up the entangled mucin matrix.

In this study, we observed a two-fold decrease of the average swimming velocity of *P. aeruginosa* with aerosolized hypertonic saline (Ctrl: 8.69 ± 1.41 µm/s; hypertonic saline: 3.5 ± 0.6 µm/s). Bacteria that were given hypertonic saline-aerosol treatment had a much broader spectrum of migration when compared to bacteria cultured in hypertonic saline alone^23^. We hypothesize that the broad speed distribution may have been interrupted by the random deposition of the aerosol, which only delivered hypertonic saline locally at the deposition sites. The motility of *P. aeruginosa* plays crucial roles in disease progression and adaptive antibiotic resistance, especially in the presence of mucin^19,24,25^. For instance, antibiotic resistance may associate with collective dysregulation of genes^23–25^. Altered motility can also attribute adaptive resistance nongenetically; faster migration enables higher adaptive resistance whereas slower bacteria endure longer antibiotic exposure^23^. Based on previous findings, the decrease in bacterial motility was speculated to contribute to the lower gentamicin tolerance with aerosolized hypertonic saline treatment. However, the underlying mechanisms of motility-mediated antibiotic resistance are complicated, and more studies that are detailed are required to further elucidate the impacts of decreased *P. aeruginosa* motility after hypertonic saline aerosol treatment.

Altered motility can also influence biofilm formation^26^. A decrease in motility may be associated with the observed unique 3-D hole structure of biofilm in the presence of gentamicin. Microbial biofilms provide a shelter for bacteria from antimicrobial agents^24^. In the optical section images, we observed hole structures extending from the colony surface to the bottom. While comparing the microstructure of colonies treated with control and saline-gentamicin aerosols, we expected the holes to be generated by the deposited hypertonic-gentamycin aerosols. The 3-D hole structures suggested that the aerosolized hypertonic saline can promote antibiotic penetration into the biofilm shelter, which supported our previous antibiotic penetration experiments. Moreover, based on the high density of dead bacteria surrounding the hole structures and motility results, we inferred that aerosolized hypertonic saline not only promoted antibiotic penetration, but also locally decreased the motility of *P. aeruginosa*, which may contribute to the elimination of antibiotic tolerance at deposited sites. In addition, the alterations in the microenvironment, such as the oxygen supply and nutrient gradient, resulting from the structural changes in the microbial biofilms, can influence the interaction with the microbial community^27^. However, further in-depth studies are needed to clarify the profound effects of microstructural changes.

Our *in vitro* experiments demonstrated the compounding effects of a hypertonic saline and antibiotic agent aerosol and elucidated the mechanism that contributed to the therapeutic efficacy. We found that hypertonic saline enhances aerosol penetration through the mucus and microbial biofilm when hypertonic saline was used as the aerosol carrier for gentamicin. Moreover, aerosolized hypertonic saline may reduce the antibiotic tolerance by suppressing microbial motility at the deposition site. In summary, our results demonstrated the utilization of low-cost and highly-safe hypertonic saline that may promote the efficacy of antibiotic aerosols, which may contribute to the current use of inhaled therapeutic compounds.

## Methods

### Measurement of nanosphere diffusivity

Porcine stomach mucin (primarily MUC5) was purchased from Sigma–Aldrich (St. Louis, MO, USA) and was used as a model for human mucin^28^. Purified mucin solutions were prepared by dissolving mucin at various concentrations in 0.9 wt% and 7 wt% sodium chloride phosphate-buffered saline. Mucin solutions were prepared 24 h prior to the experiments to ensure equilibrium. Microrheology of the mucin matrix was evaluated using a particle tracking approach. Brownian motion of carboxyl-activated polystyrene microspheres (500 nm, Polysciences, Warrington, PA, USA) in mucin solutions was recorded using a 60X oil-immersion objective and sCMOS camera (Hamamatsu, Shizuoka, Japan). Sphere trajectories were analysed and converted to mean-square displacements (MSDs) using ImageJ (National Institutes of Health, Bethesda, MA, USA) and MatLab (MathWorks, Natick, MA, USA).

### Molecule penetration in mucus matrix

We used both fluorescence-labelled dextran and gentamicin to evaluate the drug penetration in a mucin matrix. Fluorescence-labelled dextran (MW: 40kD, Sigma Aldrich, MO, USA) was used for studying the molecular diffusion in either the polymeric matrix or *in vitro* models^29,30^. Gentamicin and tobramycin are first generation aminoglycoside antibiotics used for treating upper airway infections in patients with cystic fibrosis and critically ill patients. Tobramycin inhalation solution is not marketed in some countries due to a very low population of cystic fibrosis. In contrast, gentamicin is a relatively cheap antibiotic, compared to other injectable aminoglycoside solutions; thus, gentamicin was selected for this study. Experiments were conducted using transwell membranes (Corning Inc., Corning, NY, USA) with a pore size of 4 µm. Briefly, 200 µL of prepared mucin (60 mg/mL and 30 mg/mL) was added to the upper well of the transwell support. After 2 h of incubation, 100 µL of hypertonic saline (or saline) containing gentamicin was gently added on top of the mucin layer; the diffused gentamicin was sampled at specific time points from the lower well. Dextran samples were collected and anlysed using spectrophotometer^29,30^; Gentamicin samples were collected and analysed using liquid chromatography–mass spectrometry as previously described^9^.

### Bacterial colony growth and aerosol delivery system

The *P. aeruginosa* strain PAO1 (ATCC 47085) was used. Bacteria were routinely grown in tryptic soy broth under shaking culturing at 37 °C according to ATCC protocol. The spread area assay was adapted to evaluate the effect of the aerosol on colony-like structures^31,32^. In short, 2 µL of bacterial subculture suspension (OD_600_ of 0.5, ~5 × 10^7^ CFU/mL) was inoculated onto tryptic soy agar with or without mucus (using 1% agar with 0.3% mucin surface coating); the plates were air-dried and the samples were cultured under static conditions at 37 °C. To evaluate the efficacy of aerosols and the influence of colony structure on antibiotic-tolerance, bacteria were treated with aerosols at 0 h and 8 h after being added to the agar plate. After aerosol treatment, colonies were incubated under static conditions at 37 °C for additional 24 h, respectively. Antibiotic efficacy was assessed according to the growing diameter of the colony; images were analyzed using ImageJ.

Colony formation assay was performed to evaluate the number of bacteria after treatment. In short, the colony-like structures were prepared as mentioned and were treated with aerosols at desired time-points as indicated. Then, a single colony was isolated and washed thoroughly with fresh medium. The surviving bacteria were seeded in appropriate dilutions to form colonies. After overnight incubation, the colony number was counted and the surviving bacteria number was calculated accordingly.

A 2.5-L closed aerosol delivery chamber was designed and the culture dish was set inside. The following solutions were placed in a pneumatic nebulizer (Galmed Inc., Taipei, Taiwan) powered by a 50 psi compressed oxygen flow at 6 L/min: (1) 0.9% saline, (2) 7% hypertonic saline, (3) 1.25 mg/mL gentamicin mixed with 6 mL of 0.9% saline, and (4) 1.25 mg/mL gentamicin mixed with 6 mL 7% hypertonic saline. Nebulization was stopped at sputtering and the culture dish was removed 30 s later. The aerosol particle size distribution of volume median diameter (VMD) was measured by a laser diffractometry (SprayTech, Malvern Instruments Ltd., Worcestershire, UK).

### Biofilm imaging and bacterial viability assay

A bacterial viability kit (BacLight Invitrogen; Thermo Fisher Scientific, Waltham, MA, USA) was used to determine the microbial viability. After aerosol treatment, bacteria were stained, according to the manufacturer’s instructions; the live and dead cells were determined based on different fluorescence diffused through cell membranes. Fluorescent imaging was performed using epi-fluorescent microscopy (Ti; Nikon Instruments, Tokyo, Japan) and laser scanning confocal microscopy (FV-300; Olympus, Tokyo, Japan). The overall colon shape was large-area scanned to obtain epi-fluorescent microscopy images, which were used to monitor the overall colony features; the 3-D microstructure of colonies was studied using confocal microscopy.

### Statistical analysis

All data were presented as mean ± standard deviation (SD). Comparisons among groups were conducted using one-way analysis of variance (ANOVA) with post-hoc Bonferroni correction. Student’s two-tailed t-test was applied to determine the significance of difference between the presence of mucin and without mucin, and the results were indicated as *p  <  0.05, **p  <  0.01, ***p  <  0.001.
